# A Review of Scales to Evaluate Sleep Disturbances in Movement Disorders

**DOI:** 10.3389/fneur.2018.00369

**Published:** 2018-05-29

**Authors:** Mónica M. Kurtis, Roberta Balestrino, Carmen Rodriguez-Blazquez, Maria João Forjaz, Pablo Martinez-Martin

**Affiliations:** ^1^Movement Disorders Unit, Neurology Department, Hospital Ruber Internacional, Madrid, Spain; ^2^Department of Neuroscience Rita Levi Montalcini, University of Turin, Turin, Italy; ^3^National Center of Epidemiology and Centro de Investigación Biomédica en Red sobre Enfermedades Neurodegenerativas (CIBERNED), Institute of Health Carlos III, Madrid, Spain; ^4^National School of Public Health and Red de Investigación en Servicios de Salud en Enfermedades Crónicas (REDISSEC), Institute of Health Carlos III, Madrid, Spain

**Keywords:** Parkinson’s disease, Parkinsonism, chorea, dystonia, insomnia, nocturia, restless legs, sleep apnea

## Abstract

Patients with movement disorders have a high prevalence of sleep disturbances that can be classified as (1) nocturnal sleep symptoms, such as insomnia, nocturia, restless legs syndrome (RLS), periodic limb movements (PLM), obstructive sleep apnea (OSA), and REM sleep behavior disorder; and (2) diurnal problems that include excessive daytime sleepiness (EDS) and sleep attacks. The objective of this review is to provide a practical overview of the most relevant scales that assess these disturbances to guide the choice of the most useful instrument/s depending on the line of research or clinical focus. For each scale, the reader will find a brief description of practicalities and psychometric properties, use in movement disorder cohorts and analyzed strengths and limitations. To assess insomnia, the Pittsburgh Sleep Quality Index, a generic scale, and three disease-specific scales: the Parkinson Disease Sleep Scale (PDSS), the PDSS-2, and Scales for outcomes in Parkinson’s disease (PD)-Sleep-Nocturnal Sleep subscale are discussed. To evaluate nocturia, there are no specific tools, but some extensively validated generic urinary symptom scales (the Overall Bladder Questionnaire and the Overactive Bladder Symptom Score) and some PD-specific scales that include a nocturia item are available. To measure RLS severity, there are currently four domain-specific generic scales: The International Restless Legs Scale, the Johns Hopkins Restless Legs Severity Scale, the Restless Legs Syndrome-6 measure, a Pediatric RLS Severity Scale, and the Augmentation Severity Rating Scale (a scale to evaluate augmentation under treatment) and several instruments that assess impact on quality of sleep and health-related quality of life. To evaluate the presence of PLM, no clinical scales have been developed to date. As far as OSA, commonly used instruments such as the Sleep Apnea Scale of the Sleep Disorders Questionnaire, the STOP-Bang questionnaire, and the Berlin Questionnaire are reviewed. Three scales have been extensively used to assess EDS: the generic Epworth Sleepiness Scale, the Stanford Sleepiness Scale, and the PD-specific Scales for outcomes in PD-Sleep-Daytime sleepiness subscale. To date, only the Inappropriate Sleep Composite Score specifically evaluates propensity to sleep attacks.

## Introduction

Sleep problems such as insomnia, nocturia, restless legs syndrome (RLS), periodic limb movements (PLMs), REM sleep behavior disorder (RBD), obstructive sleep apnea (OSA), and excessive daytime sleepiness (EDS) are prevalent in the general population and in movement disorder patients. Insomnia, for example, is a salient problem in Parkinson’s disease (PD) (1), multiple system atrophy, progressive supranuclear palsy, and corticobasal degeneration (2), but also in patients with hyperkinetic disorders such as Huntington’s chorea, neuroacantocytosis, and Tourette syndrome (3). Daytime sleepiness (DS) is one of the main features of Lewy body dementia and is also problematic in PD (4) and may be particularly influenced by treatment with dopamine agonists as well as levodopa.

Sleep disturbances affect quality of rest and health-related quality of life (HRQoL) and may lead to problems during the waking hours, such as EDS, fatigue, and memory and attention difficulties. Nocturnal disturbances alter blood pressure, oxygen, and carbon dioxide blood levels and are thus well-established risk factors for cardio-cerebrovascular diseases. On the other hand, DS and sleep attacks impair social and working function and are highly dangerous if subjects are driving a vehicle, with evident implications beyond the patient.

Therefore, diagnosis and treatment of sleep disorders is a priority for clinicians but also for health policy makers. Diagnosis of insomnia is based on the subject’s perception of sleep quality while diagnosis of nocturia, and RLS is based on careful clinical history and evaluation. Many instruments have been designed to detect these problems, measure their severity, evaluate their effect on quality of life, and assess their change in time or after intervention. The diagnostic criteria of other sleep disturbances, such as PLMs, RBD, and OSA, are defined by polysomnographic (PSG) data. For EDS and sleep attacks, objective tests such as the Sleep Latency Test (MSLT) and the Maintenance of Wakefulness Test (MWT) are used. PSG is not a useful ancillary test to diagnose EDS, although it can be useful to identify underlying sleep disorders (5). However, these laboratory tests are highly demanding in terms of cost, human resources, and other logistics required; therefore, questionnaires and scales have been developed for screening patients and thus make recommendations for further testing.

Questionnaires are patient-based instruments that allow for the evaluation of signs and symptoms that cannot be observed in the clinic (i.e., insomnia or nocturia) and may include subjective features based on the patient’s perception and judgment of the symptom and its impact on his/her life. As such, they are considered patient-related outcomes and have the advantage of shifting the focus to the patient, a requirement in the current biopsychosocial model of patient-centered medicine.

The objective of this review is to provide the clinician and researcher with an overview of the scales that are currently available to screen and measure the severity of these sleep disturbances (except RBD, which is the focus of another article in this issue). All the reviewed scales are questionnaires and are organized by type (generic, disease-specific, and HRQoL) and chronologically by year of publication. After a practical description of each scale including diagnostic accuracy (for screening instruments, where available), the psychometric properties and application in different movement disorder cohorts are discussed, along with the instruments’ advantages and disadvantages.

## Methods

The literature search was based on the pubmed database and included articles published before January 31, 2018 and written in English, Italian, French or Spanish. The combined MeSH search terms used were: (“insomnia” OR “nocturia” OR “restless legs” OR “periodic leg movements” OR “obstructive sleep apnea” OR “excessive daytime sleepiness” OR “sleep attacks” OR “sleep disturbance”) AND (“Parkinson’s disease”). A similar search strategy was performed with the following terms: “multiple system atrophy,” “dementia with lewy bodies,” “progressive supranuclear palsy,” “corticobasal degeneration,” “dystonia,” “chorea,” “Huntington’s chorea,” “tics,” “Tourette.” The identified articles were perused for the use of scales or questionnaires for screening or diagnosis of the sleep disturbances under review, and their references were also searched for other sleep disturbance scales. For each identified measuring instrument, psychometric properties were noted from the original developers of the instrument and completed with further clinimetric investigations when available. With all identified generic scales, a similar search strategy was used to identify their use in the different movement disorder populations mentioned above. The extracted information was initially discussed and agreed by two authors (Mónica M. Kurtis and Roberta Balestrino), and their consensus was reviewed by the other authors to obtain a final agreement of the most relevant scales.

## Nocturnal Sleep (NS) Disturbances

### Insomnia

The diagnostic criteria of “insomnia syndrome” include the following: (1) difficulty falling asleep, awakenings during the night or waking up too early, (2) despite adequate sleep circumstances, and (3) symptoms during wakefulness, such as attention problems or fatigue, due to the lack of sleep (6). Thus, the diagnosis depends on self-reported outcomes, not on a specific amount of sleep or other objective sleep measures. Insomnia can be classified as primary or secondary; however, it is often hard to establish the cause, effect, or coexistence of conditions that are associated with insomnia such as psychiatric disorders. Thus, the term “comorbid” is preferable to “secondary.” Insomnia is a frequent complaint in movement disorder patients and has been extensively studied in PD, chorea, and antibody-mediated encephalopathies manifesting with movement disorders.

#### Pittsburgh Sleep Quality Index (PSQI)

##### Scale Description

The PSQI is a generic, 19 items self-rated scale designed to measure overall sleep problems (7). A score above 5 distinguishes between “good” and “poor” sleepers with a high sensitivity and specificity (7, 8) (Table 1).

**Table 1:** Scales that evaluate insomnia in movement disorders.

Scale	Overview	Scoring	Cut off Diagnostic accuracy	Time frame	Administration	Languages
PSQI (7)	Generic Assesses overall sleep quality	19 items are combined to form seven component scores (subjective sleep quality, sleep latency, sleep duration, habitual sleep efficiency, sleep disturbances, use of sleeping medication, and daytime dysfunction). Items are scored from 0 to 3 (no difficulty to severe difficulty). Total scores range from 0 to 21, where higher scores indicating more severe difficulties in the different areas	>5 “bad sleepers” Sensitivity 90–98% Specificity 84–86% (7, 8)	Previous month	Self-rated 10 min	Public domain English Spanish
PDSS (17)	PD specific Measures nocturnal problems and daytime sleepiness	15 items rated on visual analog scale (0–10). Range 0–150, where higher scores indicate more severe sleep problems. Weighted toward severity	>82 AUC: 0.85 (95% CI 0.78–0.91) Sensitivity 75% Specificity 80% (18)	Previous week	Self-rated 10 min	Public domain English Japanese Spanish Portuguese
PDSS-2 (21)	PD specific NS problems	15 items scored from 0 (never) to 4 (very frequent). Range 0–60, where higher scores indicate more sleep problems	≥15 Sensitivity 72.1% Specificity 72.9% (21)	Previous week	Self-rated 10 min	Public domain English Spanish
SCOPA-Sleep-NS (11)	PD specific Measures NS problems and sleep quality	The NS subscale has 5 items scored from 0 (not at all) to 3 (very much). Range 0–15. Higher scores indicate more severity. One item on sleep quality is rated on 7-point scale, 0 (very well) to 7 (badly)	>7 AUC 0.96 (CI 0.93–0.98) Sensitivity 97% Specificity 80–88% (11, 18)	Previous month	Self-rated 5 min	Public domain Dutch English Spanish Thai

*AUC, area under the curve; CI confidence interval; NS, nocturnal sleep; PDSS, Parkinson Disease Sleep Scale; PSQI, Pittsburg Sleep Quality Index; SCOPA, Scales for outcomes in PD; PD, Parkinson’s disease*.

##### Psychometric Properties

The scale has shown high internal consistency and homogeneity (Cronbach’s alpha = 0.80–0.83) (7, 9). A factor scoring model based on three different domains (sleep efficiency, perceived sleep quality, and daily disturbances) is suggested (10). This scale shows strong correlation with SCOPA-Sleep but not with PSG, except for sleep latency (7, 11).

##### Use in Different Movement Disorder Populations

The scale has been extensively used in primary insomnia, dementia, depression and anxiety but also in movement disorders such as PD (12), MSA, PSP (13), and Huntington’s disease (14). It has shown sensitivity to change in PD cohorts after interventions such as deep brain stimulation (15) and pharmacological treatment (16).

##### Strengths and Limitations

The PSQI is a generic scale with strong psychometric attributes that has been used in different movement disorders populations and reliably measures overall sleep problems. The scale was “recommended” by the MDS Task Force that reviewed clinical scales assessing sleep in PD as a screening tool and a measure of severity for overall sleep problems (10). The PSQI has the limitations of a self-rated scale (unreliable in dementia populations), although it does include 5 items to be filled out by the caregiver that are not included in the total score. It is heavily weighted toward sleep habits and does not adequately cover other sleep disturbances such as motor problems at bedtime (akinesia, dystonia, or chorea), RLS, REM Sleep Behavior Disorder (RBD) or DS. Furthermore, questions addressing respiratory disturbances and awakening may be confounders since they may be secondary to different problems, and scoring is complex.

#### Parkinson Disease Sleep Scale (PDSS)

##### Scale Description

The PDSS (17) is a PD-specific, 15 item self-rated scale that preferentially evaluates NS problems, with only one item pertaining to DS [see Nocturnal Sleep (NS) Disturbances]. Each item is rated on a 0–10 visual analog scale (VAS), total score ranging from 0 to 150. A cutoff score above 82 indicates the presence of NS problems with acceptable sensitivity and specificity (18) (Table 1).

##### Use in Different Movement Disorder Populations

The PDSS has been extensively used in PD and there is some experience in a dystonia cohort including patients with generalized, segmental, and focal dystonia (19).

##### Psychometric Properties

The PDSS has been validated in PD patients in all stages (20). Internal consistency and test–retest reliability are high [Cronbach’s alpha = 0.77 and intraclass correlation coefficient (ICC) = 0.94, respectively] (17). Floor and ceiling effects are low, and there is significant correlation between 11 of the 15 items (20). The PDSS has shown strong correlation with the SCOPA-Sleep-NS (18).

##### Data From Different Populations

The PDSS has been widely used in different PD populations and distinguishes between PD patients and controls and drug naïve, mild and long-standing PD patients.

##### Strengths and Limitations

The PDSS has demonstrated good psychometric characteristics. The scale is “recommended” by the MDS Task Force as a screening tool and severity measure for sleep symptoms in PD as it includes most of the patient’s possible disturbances (10). However, patients require explanation on how to score the VAS, and the scale does not include information from the caregiver. It does not address respiratory difficulties such as sleep apnea and specific sleep disturbances such as RBD and RLS are partially addressed but wording is ambiguous. Finally, the scale only has one item regarding DS and thus is not recommended to assess diurnal sleep problems in PD.

#### Parkinson Disease Sleep Scale-2

##### Scale Description

The PDSS-2 (21) is the second version of the PDSS and has two main differences with the first version of the PDSS (see above): items are scored on a Likert scale (from 0 = never to 4 = very frequent), and all 15 items evaluate NS problems. Total scores range from 0 to 60, and higher scores indicate higher severity. A cut of score above or equal to 15 distinguishes “bad” sleepers from good sleepers with acceptable diagnostic accuracy (21) (Table 1).

##### Psychometric Properties

The scale has shown acceptable inter-item correlations (>0.30) and internal consistency (Cronbach’s alpha, 0.73). Test–retest reliability is also satisfactory (ICC = 0.80). Factor analysis resulted in a strong main factor that justifies summing all the items into a total scale, but also a three factors solution explaining 42.75% of variance, which suggested a hierarchical scale structure. The three subscales are night-time motor problems, PD-specific symptoms, and sleep-specific symptoms (21).

##### Strengths and Limitations

Like the previous version, the PDSS-2 has shown good psychometric characteristics and has been extensively used in PD and is thus recommendable for screening and measuring severity of sleep disturbances in PD. This Likert scaling is easier for patients to understand, although scoring in item 1 is inverted which may be confusing.

#### SCOPA-Sleep (NS Subscale)

##### Scale Description

The Scales for Outcomes in PD-Sleep (SCOPA-Sleep) (11) is a PD-specific scale that includes 12 items to measure sleep quality, NS disturbances, and DS. The NS subscale includes 5 items on insomnia, multiple awakenings, sleep efficiency, and duration plus one single item on overall sleep quality. A cutoff score of 7 has demonstrated excellent sensitivity and satisfactory specificity (11, 18) (Table 1).

##### Psychometric Properties

The internal consistency of the NS subscale is high (Cronbach’s alpha = 0.88–0.84), and test–retest reliability is excellent (ICC = 0.94) (11). Factor analysis revealed one factor accounting for 68.1% of the variance, thus demonstrating that this subscale measures a single construct (11). The SCOPA-Sleep-NS has shown strong correlations with other instruments such as the PSQI (*r* = 0.83) (11) and the PDSS (*r* = 0.60) (18).

##### Strengths and Limitations

The NS subscale of the SCOPA-Sleep has shown strong psychometric properties and has been extensively used, discriminating between subjects and controls and PD patients in different stages. The subscale is a “recommended” tool for screening and for measuring severity of overall sleep problems in PD (10). However, sensitivity to change has not been investigated and, similarly to the PDSS, the scale lacks specific items addressing RLS and RBD.

#### Multidomain Scales

Some multidomain PD-specific scales, such as the Non-Motor Symptom Questionnaire (NMSQuest) (22), the Non-Motor Symptom Scale (NMSS) (23), and the Movement Disorder Society Unified Parkinson Disease Rating Scale (MDS UPDRS) part 1B (24) include a single item that evaluates insomnia. The NMSS is a rater-based scale that evaluates both severity and frequency of thirty different non-motor symptoms seen in PD, considering the past month. Item 5 has been used to evaluate difficulty falling asleep or staying asleep in other hypokinetic disorders such as MSA and PSP (25). The NMSQuest and the MDS UPDRS part 1B are both self-rated questionnaires. Question 23 on the NMSQuest evaluates difficulty falling asleep or maintaining sleep while item 1.7 of the MDS UPDRS asks the patient to give an overall score on sleep quality in the past 7 days. Another disease-specific scale for another parkinsonism, the Progressive Supranuclear Palsy Rating Scale (26) includes one item on sleep difficulty, rated from 0 to 4, in the activities of daily living part. These scales have demonstrated excellent psychometric characteristics, although the validity and reliability of single items has not been tested yet.

### Conclusion

Only the PSQI can be recommended for screening and assessing severity of insomnia in any type of movement disorders since it is a generic scale with satisfactory psychometric properties and extensive use. There are three PD-specific scales that can be recommended in patients with this disease. There are single items on PD-specific multidomain scales that assess insomnia, but these single items have insufficient psychometric data on validity and reliability.

### Nocturia

Nocturia is defined as “waking up to pass urine during the main sleep period,” and it is a common phenomenon although patients tend to under-report this symptom (27). Nocturia can be due to different causes, such as renal, urological, vascular, or neurological diseases and medications, and etiology ranges widely by gender and age group. Similarly to other sleep disturbances, consequences of nocturia include lower sleep quality with eventual daytime consequences. Furthermore, nocturia is associated with falls and bone fractures, therefore increasing morbidity and, in elderly populations, mortality (27). Nocturia is highly frequent in patients with PD and can exacerbate sleep fragmentation among this population (28).

#### Overactive Bladder Questionnaire (OAB-q)

##### Scale Description

The OAB-q (29) is a generic, self-rated, 33 items scale developed to measure symptoms of overactive bladder (OAB), which include urinary frequency, nocturia, and urgency, with or without incontinence. The scale is made up of two subscales: a symptom bother scale (8 items), rated 1 (not at all) to 6 (a very great deal) and an HRQoL scale (HRQoL) (25 items), rated 1 (none of the time) to 6 (all the time). Two items of the symptom bother scale assess nocturia, and five of the HRQoL scale are related to sleep. The time frame is the past 4 weeks. It takes about 20–25 min to complete. A shorter form, the OAB-q SF, made up of 19 items (6 symptom bother items and 13 HRQoL items) was developed by the original authors and includes nocturia (1 item) and impact on sleep quality (2 items) (30).

##### Psychometric Properties

Factor analysis provided a four factors solution for the HRQoL items (one of them being sleep) (29). Internal consistency is high with subscale Cronbach’s alpha-values ranging from 0.86 to 0.94 (29). The questionnaire has also demonstrated adequate test–retest reliability (31) and satisfactory responsiveness (32). The shorter form has also shown appropriate convergent validity, discriminant validity, internal reliability, reproducibility, and responsiveness to change (30).

##### Scale Use in Movement Disorders

In PD, the OAB-q has been used to evaluate the correlation of bladder dysfunction and motor impairment (33), while its short form has been used to evaluate the effect of percutaneous posterior nerve stimulation on detrusor (34) but no published experience in other movement disorder cohorts was found.

##### Strengths and Limitations

The OAB-q has been extensively used due to its robust psychometric characteristics and because it assesses severity of symptoms and impact on quality of life. It can be found in several languages and a short form is available. However, studies demonstrating sensitivity to change of the OAB-q are lacking. The psychometric properties of the items on nocturia have not been investigated nor their impact on sleep quality. Finally, data in movement disorders patients are only available for PD patients.

#### Overactive Bladder Symptom Score (OABSS)

##### Scale Description

The OABSS (35) is another generic, self-rated scale with 4 items that include: daytime urinary frequency (scored 0–2), nocturia (from 0, never wake up to urinate, to 3, wake up ≥3 times to urinate), urgency (0–5), and incontinence (0–5). Thus, the total score ranges from 0 to 15, where higher scores indicate more severity. The time frame is the past week, and the questionnaire takes about 3–4 min to complete.

##### Psychometric Properties

In patients with OAB, each symptom score correlated positively with the total OABSS (*r*_S_ = 0.10–0.78), and the scale showed good internal consistency (Cronbach’s alpha = 0.74) and high test–retest reliability (weighted kappa coefficients were 0.80–1.0 for each symptom score and 0.86 for OABSS). The OABSS showed moderated correlations with quality of life scores assessed by the King’s Health Questionnaire (*r* = 0.20–0.49). The OABSS discriminated patients with OAB from controls and showed sensitivity to change after therapeutic intervention (35).

##### Use in Movement Disorders

The OABSS has been recently used in two PD cohorts to evaluate urinary symptoms, including nocturia. Mito et al investigated PD patients without treatment and found moderate correlations between OABSS and the UPDRS motor scores (*r*_S_ = 0.39), particularly with the akinetic-rigid subscore (*r*_S_ = 0.47) (36). Another study investigated the correlation between urinary disturbances and falls in 90 patients with PD and did not find a relationship between nocturia and falling (37). To the best of our knowledge, the scale has not been used in other movement disorders.

##### Strengths and Limitations

The OABSS is a fast and easy to use scale that has demonstrated content and construct validity and internal consistency in measuring urinary disturbances in OAB syndrome. However, the psychometric properties of the single item on nocturia have not been sufficiently investigated, and data for movement disorders populations are limited to PD.

#### Urinary Symptom Profile (USP)

##### Scale Description

The USP is a 13 item, self-rated scale that evaluates three dimensions of urinary disturbances: stress urinary incontinence, OAB, and low stream symptoms (38). The OAB domain includes two items on nocturnal urinary symptoms. The time frame includes symptoms in the past 4 weeks.

##### Psychometric Properties

The scale has demonstrated robust psychometric qualities with good internal consistency, convergent validity, and test–retest reproducibility. In the validation study, USP dimension scores were good predictors of urinary disorder presence and identification and correlated with micturition diaries (38).

##### Use in Movement Disorders

To the best of our knowledge, the scale has only been used in a population of functional movement disorders patients who reported lower urinary tract symptoms (39).

##### Strengths and Limitations

Although this is the first scale to comprehensively assess the main dimensions of urinary disturbances in both sexes and the psychometric properties are more than adequate, the UPS has scarcely been studied in movement disorders patients. The nocturia items have not been investigated separately to evaluate their psychometric properties.

#### Other Generic Urinary Symptom Scales

There are four generic questionnaires designed to assess lower urinary tract symptoms associated with benign prostatic enlargement in men that have excellent psychometric properties (validity and reliability) and include an item on nocturia. Of these, the most extensively used, probably due to simplicity and fast completion, is the American Urological Association symptom score (40), also known as the International Prostate Symptom Score. The Danish Prostate Symptom Score (41) and the International Continence Society (ICS) ICSmale questionnaire (42) are very complete but requires more time and harder to score. The shortened version of the latter, the ICSmale Short Form (43), provides a good alternative and has been used in a Huntington’s disease population (44).

#### PD-Specific Multidomain Scales

The NMSQuest (22), the NMSS (23), and the SCOPA-Autonomic (45) include an item on nocturia. Item 24 on the NMSS and item 9 on the NMSQuest evaluate whether the patient has to get up during the night to urinate. The SCOPA-Autonomic is a tool that evaluates the range of dysautonomic symptoms that most affect PD patients and item 13 evaluates the frequency of nocturia. These single items have not been properly investigated to establish their psychometric properties and reliability to screen for and measure severity of nocturia in PD.

### Conclusion

There is no scale designed solely for the evaluation of nocturia and its effect on sleep. The available measurements include generic scales that measure lower urinary tract dysfunction, including nocturia, and PD-specific scales that address a range of non-motor symptoms that are prevalent in this movement disorder, including nocturia.

### Restless Legs Syndrome

Restless legs syndrome is a neurological disorder characterized by the presence of unpleasant sensations that patients describe as creeping, crawling, itching, tingling, pulling, or painful sensations, more commonly, but not exclusively, in the lower limbs. Moving the interested limb or standing up, walking or stretching can release these sensations, thus the name of the disease. RLS is relatively common, with prevalence rates ranging from 3.9 to 15% in the general population; is more common among Caucasians and older people, although it can affect children as well (46). Approximately 10% of patients seek medical help, and the most common complaints are difficulty in sleeping/daytime activities and an overall lower HRQoL (47). The diagnostic criteria have been recently revised by the International Restless Legs Syndrome Study Group (48).

There are three scales to assess the severity of RLS, one scale to assess the augmentation phenomenon, three scales that assess HRQoL and two scales that assess the impact of RLS on sleeping and on daily functioning, although just one has been validated (Table 2). One pediatric scale has been designed but has not been validated. Two PD multidomain scales have an item to assess symptoms that are compatible with RLS.

**Table 2:** Scales that evaluate restless legs syndrome.

Scale	Overview	Scoring	Cut off Diagnostic accuracy	Time frame	Administration	Languages
JHRLSS (49)	RLS specific Assesses the usual time of onset/severity of symptoms	1 question on what time of day the RLS appears, with answers ranging from 0 (no symptoms) to 3 (day and night symptoms)	No established cutoff	Lifetime (50% of days)	Clinician rated 1 min	English
IRLS (51)	RLS specific Most used scale to assess severity of RLS	10 items in total. Answers range from “no RLS or impact (0)” to “very severe RLS or impact (4)” 2 subscales: symptoms and symptoms impact Total score that ranges from 0 to 40	No established cutoff	Previous week	Clinician rated 10 min	English, Japanese, Hindi, Brazilian Portuguese, and translations performed by MAPI Research Trust^a^
RLS-6 (55, 56)	RLS specific Assess severity of RLS at different times of a 24 h period	6 items, scored on a 0–10 scale (0 = no symptom, 10 = very severe). No total score, separate scores for 4 domains: sleep quality (items 1 + 6); RLS at night time (items 2 + 3); daytime manifestations during relaxation (item 4); and during activity (“RLS mimics”) (item 5)	No established cutoff	Previous week	Clinician rated 10 min	English
ASRS (57)	RLS specific Measures RLS before and after dopaminergic treatment	3 items cover where symptoms begin and the onset. Each item is scored “0” (improvement after treatment), worsening score ranges between 1 (“mild”) and 8 (“severe”). Total score ranges from 0 to 24 following an algorithm	≥5 Sensitivity 82% Specificity 92% (57)	Previous week	Clinician rated 10 min for the scale and 5 min to calculate the score	English and translations performed by MAPI Research Trust^b^
RLS-QLI (58)	RLS specific Measures impact of RLS on HRQoL	17 items in 4 domains: daily function, social function, sleep quality, and emotional well-being. Scores for each domain can be calculated as explained in the scale. Total scores range between 0 and 100 (lower scores mean lower HRQoL)	No established cutoff	Previous month	Self-rated 15 min for the scale and 5 min for the score	English
ARLSQoL (60)	RLS specific Measures impact of RLS on daily life, emotional well-being, social life and work	18 items. 10 items are scored on a 5-point scale, and form a single summary score, the overall life impact score (lower scores indicate worse HRQoL). The remaining 8 items are recorded as either a numerical value or a dichotomous response and concern daily activities (one question), sexual interest (two questions) and work (five questions)	No established cutoff	Previous 4 weeks	Self-rated 10 min	Dutch, Finnish, French, German, Greek, Hungarian, Italian, Hindi, and Japanese
KRLS-QoL (62)	RLS specific Measures impact of RLS on HRQoL	12 items, 5 domains (effects of RLS symptoms; disturbed sleep and its effects; effects of other features; handling the RLS symptoms; overall impact on QoL). First 11 items are scored from 0 (no impairment at all) to 5 (extreme impairment). Item 12 summarizes the impact on quality of life	No established cutoff	Previous 4 weeks	Self-rated 10 min	English
PSQ-RLS (63)	RLS specific Assesses impact of RLS on sleeping and on daily functioning	5 single item domains (overall quality of sleep, ability to function during the day, frequency of RLS symptoms, awakening at night due to RLS, length of awakening in the night due to RLS symptoms). 4 Items are assessed with a Likert scale (1–4 or 1–5); one Item is an open-ended question on the number of nights per week with RLS symptoms	No established cutoff	Previous week	Self-rated 5 min	English

*IRLS, International Restless Legs Scale; JHRLSS, Johns Hopkins Restless Legs Severity Scale; ASRS, Augmentation Severity Rating Scale; ARLSQoL, Restless Legs Syndrome Quality of Life Questionnaire/Abetz; RLS-QLI, Restless Legs Syndrome Quality of Life Instrument; KRLS-QoL, Kohnen Restless Legs Syndrome Quality of Life Questionnaire; PSQ-RLS, Post-Sleep Questionnaire for RLS; RLS-6, Restless Legs Syndrome-6; HRQoL, health related quality of life; RLS, restless legs syndrome*.

*^a^Translations: Afrikaans for South Africa, Arabic for Saudi Arabia, Cebuano for the Philippines, Czech for Czech Republic, Danish for Denmark, Dutch for Belgium (Flemish), Dutch for the Netherlands, English for Canada, English for the Philippines, English for the UK, Farsi for Iran, Finnish for Finland, French for Belgium, French for Canada, French for France, French for Switzerland, German for Austria, German for Germany, German for Switzerland, Greek for Greece, Hungarian for Hungary, Italian for Italy, Italian for Switzerland, Japanese for Japan, Korean for Korea, Mandarin for China, Mandarin for Taiwan, Norwegian for Norway, Polish for Poland, Portuguese for Portugal, Russian for Russia, Serbian for Serbia, Slovak for Slovakia, Spanish for Spain, Spanish for the USA, Swedish for Sweden, Tagalog for the Philippines, and Turkish for Turkey*.

*^b^Translations: Czech for Czech Republic, Dutch for the Netherlands, Finnish for Finland, German for Austria, German for Germany, Italian for Italy, Polish for Poland, Spanish for Spain, Swedish for Finland, and Swedish for Sweden*.

#### Johns Hopkins Restless Legs Severity Scale (JHRLSS)

##### Scale Description

The JHRLSS is a short scale used to assess the usual time of onset and severity of symptoms of RLS and consists one sole item (49) (Table 2).

##### Psychometric Properties

This scale showed a strong correlation with sleep efficiency, as assessed by an all-night polysomnogram (*r*_S_ = 0.60) and moderate correlation with Periodic Limb Movements (PLMs) per hour of sleep (*r*_S_ = 0.45). The JHRLSS inter-rater reliability was excellent: Spearman’s rank coefficient was 0.91, and Cramer’s *V* for inter-rater agreement was 0.87 (49).

##### Data From Different Populations

It has been validated in an adult population of RLS patients, with symptoms at least 5 days/week (50). We did not find any data regarding its use on other populations.

##### Strengths and Limitations

The JHRLSS is an easy and fast instrument to administer to obtain additional information on RLS. It showed correlation with objective measures of the disease. In a revision of instruments to assess the severity of RLS performed by the MDS Task Force (50), this scale was rated as “suggested” for RLS patients with frequent symptoms (5 days a week or more) since no data on its responsiveness were available. There are no published data on populations other than adults with RLS. Importantly, the demarcation point for “evening” (6:00 p.m.) might need to be adjusted based on geographic and cultural characteristics of the population. It does not provide information on other important aspects of the disease such as severity, impact on sleep, or HRQoL.

#### International Restless Legs Scale (IRLS)

##### Scale Description

The IRLS (51) is a 10-item questionnaire with two subscales, one assessing symptoms and one evaluating how bothersome they are to the patient. It is probably the most widely used tool to evaluate severity and impact on quality of life of RLS (Table 2).

##### Psychometric Properties

Factor analysis showed two factors, “Severity of symptoms” and “Life Impact,” with a total of 64.3% of the variance explained. Internal consistency was satisfactory (Cronbach’s alpha = 0.93–0.95) and corrected item-total correlations acceptable (>0.40). Reliability was adequate: after 2 weeks, the ICC was 0.87, and inter-rater reliability was 0.93–0.97. It showed strong correlation with other scales such as the Clinician’s Global Impression of Severity (CGIS) (0.73–0.74) and the Patient Global Impression (0.78–0.82). The IRLS differentiates a group of RLS patients from a normal control group and a sleep-disorder control group (51). The scale has demonstrated responsiveness (52).

##### Data From Different Populations

The scale was originally validated in an adult population of RLS patients. It has been used to study prevalence of RLS in PD and controls (53) and to compare the prevalence or RLS in different movement disorders: PD, PSP, and MSA (13). The scale has also been recently used in Huntington’s disease (54).

##### Strengths and Limitations

This scale was rated as “recommended” by the MDS Task Force (50). It is the primary instrument used to determine RLS severity, applied in different populations including movement disorder patients. It is available and validated in many different languages. However, the scale does not provide information on RLS symptom severity during different times of the day or under different circumstances.

#### Restless Legs Syndrome-6 (RLS-6)

##### Scale Description

The RLS-6 is a specific measure to assess the severity of RLS that was developed more than a decade ago (55) and has been recently validated (56). This 6 items scale is divided into four domains assessing RLS symptoms during different times and situation of the day (Table 2).

##### Psychometric Properties

The RLS-6 has adequate internal consistency (Cronbach’s alpha = 0.79). The factor analysis showed one factor explaining 55% of the variance (item 5 excluded). Moderate to high correlations were obtained between RLS-6 domains and IRLS subscores (0.25–0.70) and between RLS-6 domains and IRLS total score (0.35–0.74). The RLS-6 displayed a satisfactory ability to discriminate between patients in different severity categories as assessed by the IRLS and CGIS, and was responsive to treatment, with responsiveness coefficients ranging from 0.38 to 0.49, except for the RLS mimics domain (56).

##### Data From Different Populations

The RLS-6 has been validated in adults of both genders with RLS. To the best of our knowledge, there are no data on other populations, and this scale has not yet been used in movement disorders patients.

##### Strengths and Limitations

In a revision of instruments to assess the severity of RLS performed by the MDS Task Force (50), this scale was rated as “suggested,” since validation data had not been published. However, validation data are currently available, demonstrating good psychometric properties and good responsiveness (56). It is a complementary scale to be used with the IRLS as it provides information on symptom severity at different times of the day and night and during activities. However, there are no data on its reproducibility and stability.

#### Augmentation Severity Rating Scale (ASRS)

##### Scale Description

The ASRS was designed as a quantitative measure of the severity of augmentation (a paradoxical worsening of RLS symptoms from dopaminergic therapy) during clinical studies (57). It consists of 3 items that evaluate where (body part) and when RLS symptoms start and should be administered at baseline (before treatment) and after treatment. The scale has satisfactory sensitivity and specificity values (57) (Table 2).

##### Psychometric Properties

The ASRS showed acceptable internal consistency (Cronbach’s alpha of 0.62). Factor analysis showed only one factor. The worst augmentation score under treatment showed a strong correlation with the independent rating of an expert (0.72), and the ASRS scores were significantly different between subjects with and without augmentation according to experts’ opinion. The correlation between the CGIS for augmentation and the ASRS total score was moderate (0.53). Test–retest reliability was satisfactory (0.72); the inter-rater reliability analysis was excellent (0.94) (57).

##### Data From Different Populations

It has been validated in an adult population of RLS patients of both genders. No data on patients with other movement disorder and associated RLS are available.

##### Strengths and Limitations

The ASRS is a specific tool to measure augmentation in RLS patients who have been treated with dopaminergic agents. In the MDS Task Force review (50), this scale was rated as “recommended” to measure augmentation. The scale must be administered before and after the start of dopaminergic therapy. The main disadvantage is that it has not been correlated with objective measures of augmentation.

#### Restless Legs Syndrome Quality of Life Instrument (RLS-QLI)

##### Scale Description

The RLS-QLI is another self-administered questionnaire designed to measures the impact of RLS on HRQoL (58). This 17-item questionnaire is divided into four domains considering impact on daily activities and sleep (Table 2).

##### Psychometric Properties

Factor analysis showed 4 factors. The RLS-QLI has satisfactory internal consistency and reliability: for the subscales, Cronbach’s alpha was 0.85–0.91 test–retest reliability coefficients ranged 0.81–0.93. It showed weak to moderate correlations with SF-36 subscales (0.26–0.62) and moderate-to-strong correlations with the total IRLS score (−0.71, −0.62) (58).

##### Data From Different Populations

The RLS-QLI has been validated in adult patients of both genders, without a confirmed diagnosis of RLS. We did not find data regarding its use in other populations.

##### Strengths and Limitations

The MDS Task Force (59) rated this scale as “suggested.” It showed good psychometric properties; however, it has only been tested in a population without a confirmed diagnosis of RLS, and its responsiveness has not been assessed.

#### Restless Legs Syndrome Quality of Life Questionnaire/Abetz (ARLSQoL)

##### Scale Description

ARLSQoL (60) is a self-administered scale to measure the impact of RLS on HRQoL. This 18-item questionnaire is made up of two subscales, one evaluating overall impact and the other assessing different spheres of patients’ life (i.e., work, sexual, and social) (Table 2).

##### Psychometric Properties

There is one factor for the 10 items that are grouped in the summary score. The ARLSQoL showed excellent internal consistency (Cronbach’s alpha = 0.82–0.92) (60, 61) and satisfactory stability, with ICCs of 0.79, −0.84 (60). Correlation coefficients were moderate between the scale summary score and the Mental Component Score of the SF-36 (*r* = 0.50) and with the IRLS (*r* = −0.67 to −0.68). The ARLSQoL was able to distinguish between patients with mild, moderate or severe symptoms according to their reports, between levels of sleep problems assessed by the MOS Sleep Scale and between levels of global health status determined by a CGIS (60, 61).

##### Data From Different Populations

It has been validated in an adult population of RLS patients of both genders. To the best of our knowledge, no data on other populations are available.

##### Strengths and Limitations

This is a quick, easy and self-administered instrument to evaluate HRQoL in RLS. It has shown acceptable psychometric properties. However, its responsiveness has not been evaluated. In the MDS Task Force revision of scales to assess HRQoL in RLS (59), this scale was rated as “recommended.”

#### Kohnen Restless Legs Syndrome Quality of Life Questionnaire (KRLS-QOL)

##### Scale Description

The Kohnen Restless Legs Syndrome Quality of Life Questionnaire (KRLS-QOL) is a self-administered questionnaire to assess the HRQoL in patients with RLS. This 12-item questionnaire is divided into five domains evaluating the effect of RLS, how they are handled and overall impact (Domain 5) (Table 2) (62).

##### Psychometric Properties

The exploratory factor analysis identified two factors explaining 56.85% of the variance (“Impaired health by symptoms” and “Burden of symptoms”), but the parallel factor analysis advised to consider only one dimension in the scale.

This scale showed acceptable internal consistency and reproducibility (Cronbach’s alpha = 0.88). For test–retest reliability, kappa values were 0.43–0.64 (item 4), and the ICC for the KRLS-QoL Index was 0.73. The KRLS-QoL showed moderate-to-strong correlations with the IRLS total score (0.68) and its subscores (0.50–0.73), moderate to low correlations with the RLS-6 (0.33–0.57), and with the CGI (0.42). KRLS-QoL-Domain 5 had moderate to high correlations with the IRLS total score (0.60) and subscores (0.49–0.59), moderate to low with the RLS-6 (0.26–0.49), and CGIS (0.37). KRLS-QoL Index and Domain 5 significantly increased their scores with increasing RLS severity levels based on the IRLS and CGIS scores (62). Responsiveness parameters (effect size) showed large effect with an effective treatment, and strong correlations with change in other scales (62).

##### Data From Different Populations

The KRLS-QoL has been validated in an adult population of RLS patients of both genders. No data on other populations are available.

##### Strengths and Limitations

In the review by the MDS Task Force (59), this scale was rated as “suggested” since the validation study had not been published. However, the KRLS-QoL has now been validated and demonstrated good psychometric properties (62), therefore it can be considered a “recommended” instrument to assess the impact of RLS on HRQoL and to measure responsiveness to therapy.

#### Post-Sleep Questionnaire for RLS (PSQ-RLS)

##### Scale Description

The PSQ-RLS is a self-administered questionnaire to assess the impact of RLS on sleep and daily functioning (63). This 5-item questionnaire considers number and length of night-time interruptions of sleep due to RLS (Table 2).

##### Psychometric Properties

The PSQ-RLS showed a weak to moderate convergent validity with the following related scales: the IRLS (51), the RLSQoL (61), the Profile of Mood States (64), and sleep domains of the MOS Scale (65). In a study, PSQ-RLS scores did not include systematic measurement errors associated with personal attributes (race, gender, and ethnicity), had adequate discriminate validity across RLS severity groups, and showed satisfactory responsiveness in a 3-month treatment period (63).

##### Data From Different Populations

The PSQ-RLS has been validated in an adult population of RLS patients of both genders. No data on other populations are available.

##### Strengths and Limitations

The PSQ-RLS is an instrument that can be used to assess the impact of RLS on quality of sleep. A review of instruments that assesses HRQoL in RLS (59) noted that the scale had not been used by other investigators beyond its designers and its internal consistency and stability had not been explored, which justified the scale’s rating as “listed.” Subsequently, the scale has been used by other investigators, but the missing psychometric properties have yet to be published.

#### Restless Legs Syndrome-Next Day Impact Questionnaire (RLS-NDI)

The RLS-NDI is a self-administered questionnaire to assess the impact of RLS on sleep and daily functioning. The RLS-NDI is composed of 14 items rated on an 11-point Likert-type scale. It is designed to be administered in the evening with a 12-h recall, to assess the impact of RLS on the same day. It has shown a good content validity, but no further data on its psychometric properties are available (66).

#### Pediatric Restless Legs Syndrome Severity Scale (P-RLS-SS)

The P-RLS-SS was designed to measure RLS severity in children (67). This scale is composed of 41 items (17 morning and 24 evening items) and a parent questionnaire composed of 20 items. Its importance relies in being the only instrument to assess RLS severity in children; however it has not been validated, and no data on its psychometric properties are available.

#### Multidomain Scales That Assess RLS

For PD, there are two multidomain scales that assess the range of non-motor symptoms that are prevalent in this disease, the NMSS (23) and the NMSQuest (68). Both instruments include one item for RLS assessment when the patient is lying down or inactive: item 6 of the NMSS and item 26 of the NMSQuest.

### Conclusion

There are two scales (IRLS and RLS-6) that can be recommended to assess the severity of RLS, since they have the appropriate psychometric properties and have been extensively used. There is a recommended scale (ASRS) to assess the augmentation phenomenon. Two scales (KRLS-QoL and ARLSQoL) can be recommended to assess HRQoL in RLS. There is currently no recommended scale to evaluate RLS in the pediatric population. There are single items on PD-specific multidomain scales that assess RLS but there is insufficient evidence to evaluate their psychometric reliability or clinical applicability. Just one of the discussed scales (IRLS) has been used in several movement disorders.

### Periodic Leg Movements (PLMs)

PLMs are sleep-related movements characterized by a stereotyped and periodic pattern. They are relatively common in the general population, particularly in the elderly (69) and can occur in isolation or be associated with RLS or other movement disorders (70). The golden standard to diagnose PLMs is overnight PSG, and two sets of standards for recording and scoring are available: one by the International Restless Legs Syndrome Study group and the World Association of Sleep Medicine (71); the other by the American Academy of Sleep Medicine (72). As PSG is a cumbersome and costly test, alternative assessment tools are currently under evaluation: leg-worn actigraphy (73), contactless devices to measure movement and respiration during sleep (74) and analysis of electrocardiographic data (75) among others. Currently, there is no available questionnaire or scale to measure PLMs.

### Obstructive Sleep Apnea

Obstructive sleep apnea is a common sleep-related disorder characterized by repetitive episodes of complete or partial collapse of the upper airway which cause breath cessation. Despite being frequently underdiagnosed, its prevalence is reported to be 6–17%, varying according to age, sex, and body mass index (BMI). The gold standard for diagnosis is based on PSG recording of events of apnea (breathing pauses lasting 10 s or more) and hypopnea (reduction in respiratory airflow, without apnea, associated with oxygen desaturation or arousal from sleep). The apnea-hypopnea index (AHI) per hour is calculated to determine the presence and severity of OSA (based on standard scoring: AHI > 5 mild, AHI > 15 moderate, AHI > 30 severe) (76).

There are three questionnaires that have been designed as screening tools for OSA. The severity of OSA can be assessed by two extensively used generic scales for sleep disorders, and two specific scales have been developed to assess HRQoL in OSA. Scales that assess OSA are summarized in Table 3.

**Table 3:** Scales that evaluate obstructive sleep apnea.

Scale	Overview	Scoring	Cut off Diagnostic accuracy	Time frame	Administration	Languages
WSQ (77)	Generic on sleep disorders Investigates snoring, obstructive apnea, and sleeping problems	32 items. 10 questions on sleep disorders due to breathing, 5 questions on sleep disorders, 5 questions on medical history, and 12 questions on life habits. Multiple-choice responses with different scoring based on the question	Score of >3 point for snoring or choking (78) In general population *Any OSA stage* Sensitivity 79–95% Specificity 46–64% PPV 46–28% NPV 89–97% *Moderate OSA* Sensitivity 87% Specificity 40% PPV 11% NPV 97% (80)	3 months (81)	Self-rated 30 min	French, English, and Polish Scoring instructions provided by authors (81)
SA-SDQ (82)	Generic scale subscale Assesses sleep disturbances due to sleep apnea and sleep apnea risk factors	8 questions about sleep disturbances and 4 other items related to weight, smoking status, age, and body mass index (BMI). Each question is scored on a scale 0–5 (0 = never, 5 = always); the total score ranges 0–60	In sleep clinic patients 36 for men 32 for women Sensitivity 85–88% Specificity 76–81% PPV 31–72% NPV 87–99% (82)	Lifetime	Self-rated 8 min	English and Dutch
MAP index (84)	OSA specific Screening questionnaire for OSA	3 frequency questions (loud snoring, snoring or gasping, cessation of breathing, or struggle for breath) and gender, age, and BMI are calculated. Formulas are explained in the reference. MAP index ranges between 0 and 1	In sleep clinic patients *Mild OSA [apnea-hypopnea index (AHI) *=* 5]* Cutoff 0.46 Sensitivity 76.8% Specificity 71.8% *Moderate OSA (AHI *=* 15)* Cutoff 0.48 Sensitivity 83.3% Specificity 64.3% *Severe OSA (AHI *=* 30)* Cutoff 0.65 Sensitivity 61.8% Specificity 79.2% (85)	Last month and lifetime risk factors	Self-rated 5 min for the scale and 5 min for scoring	English
Berlin questionnaire (87)	OSA specific Screening questionnaire for OSAS	10 items, 3 domains: snoring severity, excessive daytime sleepiness, history of high blood pressure or obesity. Multiple-choice questions, for each question there is different scoring. Categories 1 and 2 are positive if total score is ≥2; Category 3 is positive if high blood pressure or if BMI > 30 kg/m^2^. Scoring: high risk: ≥2 categories with positive score; low risk: 1 or no categories with positive score	High risk score in sleep clinic patients Sensitivity 79–82% Specificity 32–39% (88)	Lifetime	Self-rated 10 min	English Arabic, Chinese, Dutch, French, Greek, Indian, Korean, Malay, Persian, Portuguese, Serbian, Thai, and Turkish
STOP-BANG questionnaire (94)	OSA specific Screening questionnaire for OSAS	There are four items on symptoms (STOP: *S*noring, *T*iredness, *O*bserved apnea, and high blood *P*ressure) and four demographic items (Bang: *B*MI, *a*ge, *n*eck circumference > 17"/43 cm in male or >16"/41 cm in female, *g*ender) *Yes/no format. Scoring* – Low risk of OSA if “Yes” to 0–2 questions – Intermediate risk if “Yes” to 3–4 questions – High risk if “Yes” to 5–8 questions or “Yes” to 2 or more of 4 STOP questions + male gender/BMI > 35 kg/m^2^/neck circumference	>3 In sleep clinic patients *For any OSA* Sensitivity 90% Specificity 49% NPV 46% PPV 91% *For moderate OSA* Sensitivity 94% Specificity 34% NPV 75% PPV 72% *For severe OSA* Sensitivity 96% Specificity 25% NPV 90% PPV 48% (99)	Lifetime	Self-rated 5 min	English and^a^
SAQLI (101)	OSA specific Measures impact on HRQoL of OSA	40 or 45 items and 4 or 5 domains: daily functioning (11 items), social interactions (13 items), emotional functioning (11 items), symptoms (five items). The 5th domain, treatment-related symptoms (5 items), can be added for adverse events of treatment. Items are scored with a Likert scale 0- to 7-point scale: “all the time” to “not at all.” Score ranges from 0 to 280/315	No established cutoff	4 weeks	Clinician rated 40–45 min	English, Spanish, Persian Portuguese, and Japanese
QSQ (104)	OSA specific Measures impact on HRQoL of OSA	32 items; five domains: (1) hypersomnolence; (2) diurnal symptoms; (3) nocturnal symptoms; (4) emotions; and (5) social interactions. Each item is scored on a 0–7 scale. Mean score per item within each domain, equal weighting	No established cutoff	Not specified	Self-rated 30 min	English, French, Chinese, Spanish, Brazilian, and Portuguese

*SA-SDQ, Sleep Apnea Scale of the Sleep Disorders Questionnaire; WSQ, Wisconsin Sleep Questionnaire; MAP, Multivariable Apnea Prediction; SAQLI, Calgary Sleep Apnea Quality of Life Index; QSQ, Quebec Sleep Questionnaire; HRQoL, health related quality of life*.

*^a^Chinese, Persian, Portuguese, Greek, French, Spanish, Afrikaans, Arabic, Bulgarian, Chinese, Czech, Dutch, Filipino, German, Hungarian, Italian, Korean, Malay, Polish, Romanian, Sami, Taiwanese, Turkish, and Arabic*.

#### Wisconsin Sleep Questionnaire (WSQ)

##### Scale Description

The WSQ is a generic questionnaire to investigate snoring, obstructive apnea, and sleeping problems in general (Table 3) (77, 78). It is one of the most cited questionnaires for OSA (79). It has a high sensitivity and a low specificity, especially for diagnosing moderate OSA, and an excellent negative predictive value (Table 3) (80).

##### Psychometric Properties

The internal consistency in each domain of the scale was satisfactory (Cronbach’s alpha = 0.67–0.81), reliability was acceptable: at retest after 3 months, the kappa values were 0.28–0.60, the Cohen kappa was >0.60 (81).

##### Data From Different Populations

This scale has been used in the general population and in a sleep disorders population. It has not been used in movement disorders to the best of our knowledge.

##### Strengths and Limitations

This is a widely used scale for assessing different aspects of OSA. It has demonstrated robust psychometric properties with a high sensitivity. However, its specificity is low, and it has not been used in movement disorders patients.

#### Sleep Apnea Scale of the Sleep Disorders Questionnaire (SA-SDQ)

##### Scale Description

The SA-SDQ is part of a generic questionnaire on sleep disorders, the Sleep Disorders Questionnaire. This subscale includes questions about sleep disturbances and demographic data. The sensitivity and specificity of this scale are acceptable, the negative predictive value was high (Table 3) (82).

##### Psychometric Properties

The scale has shown good internal consistency: Cronbach’s alpha was 0.85, and the item-total correlation ranged from 0.19 to 0.71. The intercorrelations with other subscales from the SDQ were weak (<0.35), and the scale could distinguish patients with sleep apnea from patients with other sleep disorders (narcolepsy, periodic limb movements, and psychiatric sleep disorder). The test–retest reliability after 4 months was acceptable (0.84) (82).

##### Data From Different Populations

The SA-SDQ has been validated in both genders, in patients with sleep disorders and with epilepsy (83). To the best of our knowledge, there are currently no studies that report its use in movement disorders populations.

##### Strengths and Limitations

This scale has satisfactory psychometric properties. It accurately diagnoses patients with sleep apnea events and sleep apnea related conditions. However, no data were found on the responsiveness of this scale and its use in movement disorders.

#### Multivariable Apnea Prediction (MAP) Index

##### Scale Description

The MAP index is a specific instrument used for screening sleep apnea (84) (see Table 3 for details). There is also an objective MAP index, only based on symptom frequency. The index showed acceptable sensitivity with lower specificity for detecting any OSA, and good specificity with poorer sensitivity for detecting severe OSA (85) (Table 3).

##### Psychometric Properties

The MAP index showed a moderate correlation with the AHI (*r* = 0.59). The objective MAP showed a strong correlation with the MAP index, but a poorer correlation with the AHI (85). The MAP index showed good internal consistency (Cronbach’s alpha = 0.88–0.93), and satisfactory test–retest reliability (84).

##### Data From Different Populations

This scale has been used in adults of both genders in a sleep disorder population. To the best of our knowledge, it has not been used in movement disorders.

##### Strengths and Limitations

This is a quantitative index to assess the risk of OSA. It has shown good psychometric properties. However, its use is limited since it is not useful in patients with a BMI > 40 or with mild cognitive impairment (84, 86), and there is no experience in movement disorder populations.

#### Berlin Questionnaire

##### Scale Description

The Berlin questionnaire (87) is a specific self-administered measure used to screen for sleep apnea that includes 10 items that can be divided into 3 domains. This instrument has shown adequate sensitivity but very low specificity (88) (Table 3).

##### Psychometric Properties

This questionnaire has shown satisfactory internal consistency in validation studies for different languages and populations (Cronbach’s alpha = 0.68–0.98) and acceptable test–retest reliability 0.74–0.98 (89–92). In the original validation study, the internal consistency was adequate (Cronbach’s alpha = 0.86–0.92) (87).

##### Data From Different Populations

The Berlin questionnaire has been validated in different populations including sleep clinic patients, patients before surgery, patients with cerebrovascular diseases/risk factors, multiple sclerosis patients, general population and others (89, 91, 93). It has also been used in movement disorders populations, in particular: PD, PSP, MSA (13), and Huntington’s disease (54).

##### Strengths and Limitations

The Berlin questionnaire is an easy and short instrument to screen for OSA. It has been widely used in different populations, including movement disorders populations. It has been translated into different languages. Its limitations include low specificity.

#### STOP-Bang Questionnaire

##### Scale Description

The STOP-Bang questionnaire is a specific self-administered questionnaire for screening OSA that includes questions about four symptoms and demographic data (94) (Table 3). Other versions of this scale are available: there is a shorter version (4-item STOP Questionnaire), a weighted version (wSTOP-Bang) and a continuous version (cSTOP-Bang) (95).

##### Psychometric Properties

In the original validation of the questionnaire, internal consistency was not assessed because the four questions STOP reflected four different dimensions of OSA morbidity (94). However, other investigators validating language version found moderate internal consistency (Cronbach’s alpha ranging from 0.62 to 0.7) (96, 97). Test–retest reliability has been adequate (97) and high (94, 98). The STOP-Bang questionnaire has an excellent sensitivity, but a low specificity; showing high positive and negative predictive values (99) (Table 3).

##### Data From Different Populations

The STOP-Bang questionnaire has been validated in adult patients in both genders; it has been used to screen for OSA in preoperative clinics, sleep clinics, workers at risk, kidney failure patients and in the general population (99), but, to the best our knowledge, not in movement disorders.

##### Strengths and Limitations

The STOP-Bang questionnaire is an easy and short measure to screen for OSA. It has been extensively used in different populations and has been translated into numerous languages. It is highly sensitive, but not specific; specificity can be raised using the continuous version (cSTOP-Bang), adding more variables (e.g., serum bicarbonate level) or modifying the cutoff; however, in the latter case, sensitivity drops drastically (95, 99, 100).

#### Calgary Sleep Apnea Quality of Life Index (SAQLI)

##### Scale Description

The Calgary SAQLI is a specific, self-administered scale used to measure HRQoL in patients affected by sleep apnea (101). It is a long questionnaire (40 or 45 items, if the examiner wants to assess treatment-related symptoms) (Table 3).

##### Psychometric Properties

The scale has satisfactory internal consistency (Cronbach’s alpha = 0.88–0.92 for the total scale and 0.70–91 for the subscales) and high reliability (ICC after 2 weeks = 0.92). The SAQLI showed no correlation with the respiratory disturbance index, a measure of severity of OSA, a weak correlation (−0.26) with the Epworth Sleepiness Scale (ESS), and moderate-to-strong correlations (0.39–0.71) with the sleep domains of the SF-36 (102). The SAQLI showed satisfactory responsiveness (103).

##### Data From Different Populations

This scale has been used in adults of both genders in a sleep disorder population. It has not been used in movement disorders, to the best of our knowledge.

##### Strengths and Limitations

This is a specific scale for assessing HRQoL in OSA. It offers a global evaluation of aspects of life on which OSA can impact, it has good psychometric properties, and it has been translated into different languages. However, this scale showed poor correlation with other measures of sleep apnea, sleep, and quality of life.

#### Quebec Sleep Questionnaire (QSQ)

##### Scale Description

The QSQ (104) is a specific, self-administered questionnaire that evaluates HRQoL in patients with OSA through 32 questions (Table 3).

##### Psychometric Properties

Internal consistency and reliability were satisfactory: Cronbach’s alpha was 0.76–0.94, and the ICC after 7 months ranged from 0.82 to 0.91 in the domains. It showed moderate-to-strong convergent validity with the following scales: Functional Outcomes in Sleep Questionnaire (FOSQ), Symptom Checklist-90, and ESS (−0.64). There was a high correlation with the Beck Depression Inventory (BDI) (104). The scale responsiveness has been assessed in an OSA population undergoing CPAP treatment (104).

##### Data From Different Populations

The QSQ was validated in a sleep clinic population, in adult patients of both genders. It has not been used in movement disorders to the best of our knowledge.

##### Strengths and Limitations

The QSQ is a fast and specific tool to assess HRQoL in OSA; it showed satisfactory psychometric properties and responsiveness. However, it showed a high correlation with the BDI, which can be a confounding factor. It has not been used in movement disorder patients.

#### Multidomain Scales That Assess OSA

The PSQI is a self-rated scale designed to measure generic sleep disturbances (7); items 5d and 5e of the PSQI assess, respectively, the inability to breathe comfortably and the occurrence of loud coughs or snores during sleep in the past month.

### Conclusion

The Berlin questionnaire is the only scale that has been used in movement disorders populations. This questionnaire has been validated, has shown good psychometric properties, and has been widely used. The STOP-Bang questionnaire has similar characteristics but has not been used in movement disorders. Good psychometric properties have also been identified in another screening questionnaire (MAP index) and severity assessment tools such as the WSQ, SA-SDQ. Both the SAQLI and the QSQ are validated specific questionnaires to assess HRQoL in OSA, but they have not been used in movement disorders.

## Diurnal Sleep Disorders: EDS and Sleep Attacks

Other sleep disturbances found in movement disorders affect the waking hours, such as EDS and sleep attacks. EDS is a symptom rather than a primary disease and can be the consequence of sleep deprivation, sleep disturbances as discussed above, medications or substance abuse, metabolic, neurological and psychiatric diseases or, more rarely, narcolepsy. In PD, for example, the pathophysiology of EDS is multifactorial and related to NS disturbances, other causes of sleep fragmentation (motor symptoms, painful dystonia, and dyskinesias), the neurodegenerative process itself and to medications. In fact, dopaminergic agents can disrupt sleep and induce somnolence, and sedation and drowsiness have been reported as adverse events of dopamine agonists (105).

Excessive daytime sleepiness and sleep attacks can be reported by patients or their relatives and can be assessed with self-reported scales or with objective tests such as the MSLT and the MWT. Since these laboratory tests are time consuming and costly, scales have been developed to detect and evaluate the severity of EDS and sleep attacks.

### Stanford Sleepiness Scale (SSS)

#### Scale Description

The SSS is a generic scale that measures the current state of sleepiness (106, 107). It is based on a single item rated on a Likert-type scale from 0 to 7, with higher scores indicating more subjective sleepiness.

#### Psychometric Characteristics

Data on the validity and reliability of the scale are sparse (108). The original publication claims high reliability (106). In healthy subjects, sensitivity to change has been reported (109, 110). The scale shows no correlation with the ESS which may be expected as the measured constructs are different.

#### Scale Use in Movement Disorders

The scale has been used to assess the induction of sleep by levodopa in PD versus MSA (111) and diminished homeostatic sleep drive in PSP (112). To the best of our knowledge, there are no available data in other movement disorders.

#### Strengths and Limitations

The SSS is an extensively used scale due to its availability in many languages and simplicity. It is useful for rating the sleepiness state of the individual at the time of testing. The MDS Task Force on sleep disturbances in PD concluded that the SSS is a “suggested” scale for rating sleepiness and to measure severity at a specific moment (10). However, an obvious limitation is the lack of data on psychometric characteristics of the scale.

### Epworth Sleepiness Scale

#### Scale Description

The ESS (113) is a generic scale that measures the risk of falling asleep during daily activities. This scale evaluates the possibility of dozing off in 8 every-day situations. Several cutoff scores with different sensitivities and specificities have been proposed to indicate subjects at higher risk of falling asleep involuntarily (114) (Table 4).

**Table 4:** Scales that evaluate excessive daytime sleepiness in movement disorders.

Scale	Overview	Scoring	Cut off Diagnostic accuracy	Time frame	Administration	Languages
SSS (106)	Generic Measures current state of sleepiness	1 item. Seven point Likert-type scale	No established cutoff	At this time	Self-rated	Public English French Spanish
ESS (113)	Generic Measures sleep propensity in daily situations	8 items, rated 0 (would never doze) to 3 (high chance of dosing). Range of total score 0–24	>7 Sensitivity 75% Specificity 50% >10 Sensitivity 52% Specificity 72% (115)	Recent times (1–4 weeks)	Self-rated 8 min	Public English German Spanish Chinese
ISCS (114)	Generic Measures sleep propensity and sleep attacks in active tasks	6 items, rated 0 (would never doze) to 3 (high chance of dosing). Range 0–18. Two additional questions regarding sudden sleep onset and blank spells	>1	Since disease onset	Externally rated 10 min	English
SCOPA-Sleep-DS (11)	PD specific Measures sleepiness and possibility of sleep attacks in daily activities	DS includes 6 items scored from 0 (never) to 3 (often). Range 0–18	>4 Sensitivity 90% Specificity 82%	Previous month	Self-rated	Public Dutch English Spanish

*DS, daytime sleepiness; ESS, Epworth Sleepiness Scale; ISCS, Inappropriate Sleep Composite Score; SCOPA, Scales for outcomes in PD; SSS Stanford Sleepiness Scale; SCOPA-Sleep-DS, SCOPA-Sleep Daytime Sleepiness; PD, Parkinson’s disease*.

#### Psychometric Properties

Internal consistency of the ESS is high (Cronbach’s alpha = 0.88), and the scale has demonstrated acceptable reliability (*r* = 0.56). Floor and ceiling effects are practically absent (115). ESS shows adequate convergent validity with sleep latency measured during the MSLT and during overnight PSG (115), as well as SCOPA-Sleep Daytime Sleepiness (SCOPA-Sleep-DS) subscale scores in the PD patients (11).

#### Scale Use in Movement Disorders

The ESS has been extensively used in populations with diverse sleep disorders, such as sleep apnea, narcolepsy, and idiopathic hypersomnia (113, 115) and idiopathic RBD (116). It also has been widely used in PD (10) and in other hypokinetic disorders such as MSA (117, 118). In addition, there is experience with this scale in hyperkinetic disorders such as Huntington’s disease (14, 119, 120), dystonia (19, 121, 122), and essential tremor (123–125).

#### Strengths and Limitations

The ESS has adequate psychometric properties and has been extensively used by many groups in diverse movement disorder cohorts to evaluate sleep propensity. It is recommended for screening and evaluating severity of DS in the PD population (10). It has also shown sensitivity to change after intervention. However, the scale does not include information from a caregiver, partner or other outside source; and thus, the information provided may be limited, possibly underestimating risk, since the patient may often be unaware of dozing off. The item “while in the car” is ambiguous since it does not specify whether the person is in the driver or passenger’s seat. The EES does not include an item on risk of falling asleep while driving nor does it screen for the risk of sudden sleep attacks.

### Inappropriate Sleep Composite Score (ISCS)

#### Scale Description

Two modified versions of the ESS were proposed by the Canadian Movement Disorders Group to fill the gaps of the previous scale, thus developing the ISCS (114). This scale is made up of 6 items, two from the ESS and four additional new items. If sleepiness is present, the patient is asked if dozing off occurs gradually or unpredictably and about the incidence of blank spells.

#### Psychometric Properties

The scale was developed with the objective of evaluating the predictors for sudden-onset sleep, particularly while driving, among patients with PD. It was tested in 638 mild PD patients of whom 420 were currently drivers. There was a high floor and no ceiling effect. Unfortunately, the original article does not provide any validation or reliability data and the scale has not been investigated (although has been used) by other groups.

#### Strengths and Limitations

The ISCS assesses the propensity of falling asleep during clearly active situations, such as talking or driving. The MDS Task Force reviewing sleep scales in PD gave this scale the rank of “suggested” to evaluate DS and sleep attacks and recommends its use in conjunction with the ESS (10). The scale has no published psychometric analysis, and terminology may lead to confounders since “sudden blank spells” may be due to sleep attacks, but also to syncope or partial seizures.

### SCOPA-Sleep-Daytime Sleepiness subscale

#### Scale Description

The DS subscale of the SCOPA-Sleep scale (11) includes 6 items on unexpected sleep attacks, dozing off in daily situations and difficulty staying awake. Items are scored from 0 to 3, with total scores ranging from 0 to 18 points where higher scores indicate more severe problems.

#### Psychometric Properties

The internal consistency of the SCOPA-Sleep-DS subscale is good (Cronbach’s alpha = 0.91–0.75) (11, 18). The subscale showed robust test–retest scores (ICC = 0.89) (11). Factor analysis revealed that one factor explains 69.1% of the variance for this subscale. Scores of the SCOPA-Sleep-DS have shown high correlations with the ESS (*r* = 0.81, *p* < 0.001) (11).

#### Strengths and Limitations

The DS subscale of the SCOPA-Sleep has strong psychometric properties and has been extensively used, discriminating between subjects and controls and PD patients in different stages. The subscale is a “recommended” tool for screening and for measuring severity of DS and sleep attacks in PD (10). However, sensitivity to change has not been investigated.

### PDSS Item 15

#### Scale Description

The PDSS (17) is a PD-specific, 15-item scale that preferentially evaluates NS problems, with one item pertaining to DS: item 15 (Table 1).

#### Use in Different Movement Disorder Populations

Besides being extensively used in PD patients, there are also some available data in dystonia patients (19).

#### Psychometric Properties

Item 15 on the PDSS considers DS. This single item has shown a strong correlation with SCOPA-Sleep-DS (18) and the ESS in one study (17) and a moderate correlation (0.23) in another study (20). Patients scoring low on this item (mean 4.7) have shown abnormal sleep patterns in overnight PSG results (126).

#### Strengths and Limitations

This single item has shown strong correlations with longer DS questionnaires and may thus be useful for screening for DS. However, other psychometric properties and diagnostic accuracy have not been tested.

### Multidomain Scales to Assess DS

Some multidomain scales such as the NMSQuest (22), the NMSS (23), and the MDS UPDRS part 1B (24) include a single item that evaluates DS. Item 3 on the NMSS, similarly to item 22 on the NMSQuest, evaluates if the patient dozes off unintentionally during daytime activities such as during meals or watching TV. Item 1.8 is the second item of part 1B on the MDS UPDRS considering non-motor symptoms of daily life. The patient indicates whether he/she has had trouble staying awake during the day in the last week. The limitations to the use of these single items are due to unknown psychometric properties, including lack of data regarding correlation with other scales and sensitivity to change.

## Discussion

Sleep disorders are receiving increased attention in movement disorders due to the high impact on patient HRQoL and caregiver burden as well as the growing knowledge of the underlying pathophysiological mechanisms. There are currently multiple questionnaires that can be used to screen and measure the severity of the most frequent sleep problems that affect movement disorder patients, and they have been summarized in this review. Selection of the most appropriate instrument for assessment of insomnia, nocturia, RLS, OSA, and EDS should be guided by the appropriateness of the scale for the objective of the study, availability, psychometric attributes, responsiveness, and previous experience in similar populations. In interpreting the results of questionnaires, the clinician should consider the presence of psychological factors like fatigue, impulsivity or depression that may influence completion, the timing of administration of the scale (for example, on or off state for PD patients) and cognitive impairment (127).

Several gaps have been identified that need to be addressed to adequately research the incidence, prevalence, and severity and impact of sleep disorders in populations with movement disorders.

The majority of available questionnaires are generic instruments with established psychometric properties in the general population but no validity or reliability data available in different movement disorder populations. Generic scales have the disadvantage of potentially not assessing areas of specific interest and may lack sensitivity to detect change in a given disease (128). For example, in evaluating insomnia, the well-established PSQI is currently the best option for patients with movement disorders other than PD (where disease-specific scales are available). However, it does not consider any motor symptoms, such as chorea, dystonia, tics, and other hyperkinetic movements, as well as sensitivity disturbances, such as akathisia, or RLS, that may prevent patients from falling asleep. The paucity of data available in different movement disorders populations for these generic scales is striking. For example, the prevalence of RLS in PD is still controversial due to confounders and overlap of symptoms such as dystonia, akathisia, and sensory symptoms. Validation data of the RLS scales in PD, and other forms of parkinsonism, would be helpful to decide if the current instruments are valid tools or additional measures need to be designed taking into account disease-specific symptoms that may mimic RLS. RLS is probably more frequent in ET (129) and cranio-cervical dystonia (130) than controls; however, none of the current scales have been validated in these populations.

Even in sleep disorders such as insomnia, where there are disease-specific scales for PD that are recommendable, none of them appropriately evaluate common disturbances associated with this disease such as PLMs, or sleep apnea, nor do they address motor akinesia or motor fluctuations during sleep. Most of the available scales do not consider the input of a witness, which is highly valuable in sleep disturbances, since self-perception has been reported to differ from laboratory evidence (131) and neuropsychiatric difficulties may make the patient unreliable.

For RLS evaluation, several unmet needs should be considered. To date, none of the scales that measure severity are self-administered (unlike those that consider impact on HRQoL), and the scale designed to evaluate the pediatric population still lacks psychometric data, and validity data in different movement disorders are sparse.

Currently, there are no questionnaires or scales designed to address some common sleep disorders in movement disorders such as sun-down confusion, disordered breathing and PLM. Nocturia is one of the most prevalent and bothersome symptoms for patients, yet it is only considered by 1–2 items in multidomain scales that have not been individually analyzed for their psychometric properties. Similarly, psychometric data on the scales that screen or evaluate the severity of sleep attacks, which can be potentially lethal if the person is driving, manipulating machinery or using a cutting tool, are lacking.

## Conclusion

To advance in the research of sleep and movement disorders, scientific methodology based on adequate diagnosis is of key importance. This review covers a number of questionnaires that can aid in the screening and appraisal of the most frequent nocturnal and diurnal sleep disturbances that affect movement disorder patients. However, disease-specific scales are only available for PD and even in this “best case scenario,” some prevalent symptoms such as sleep apnea or nocturia are insufficiently addressed. Thus, we recommend the development of movement disorder, condition-specific scales addressing the most important and characteristic sleep disturbances and their correlates as well as the revision of existing movement disorder scales, such as the Unified Multiple System Atrophy Rating Scale or the Unified Huntington’s Disease Rating Scale, to add a sleep domain. We also advocate for the development of guidelines to steer adequate application of existing instruments, including interpretation of results and significant change due to treatment or other causes, with the objective of improving the management of sleep disturbances in each movement disorder entity. They should include a recommended combination of assessments using different raters (health professional, patient, and caregiver) as well as advice for when further laboratory testing (PSG or MSLT) should be performed.

## Author Contributions

MK: conception and design, drafting of manuscript, interpretation of data, and revision of the manuscript. RB: drafting of manuscript and interpretation of data. CR-B and MF: interpretation of data and critical revision of the manuscript. PM-M: conception and design, interpretation of data, and critical revision of the manuscript.

## Conflict of Interest Statement

The authors declare that the research was conducted in the absence of any commercial or financial relationships that could be construed as a potential conflict of interest.
